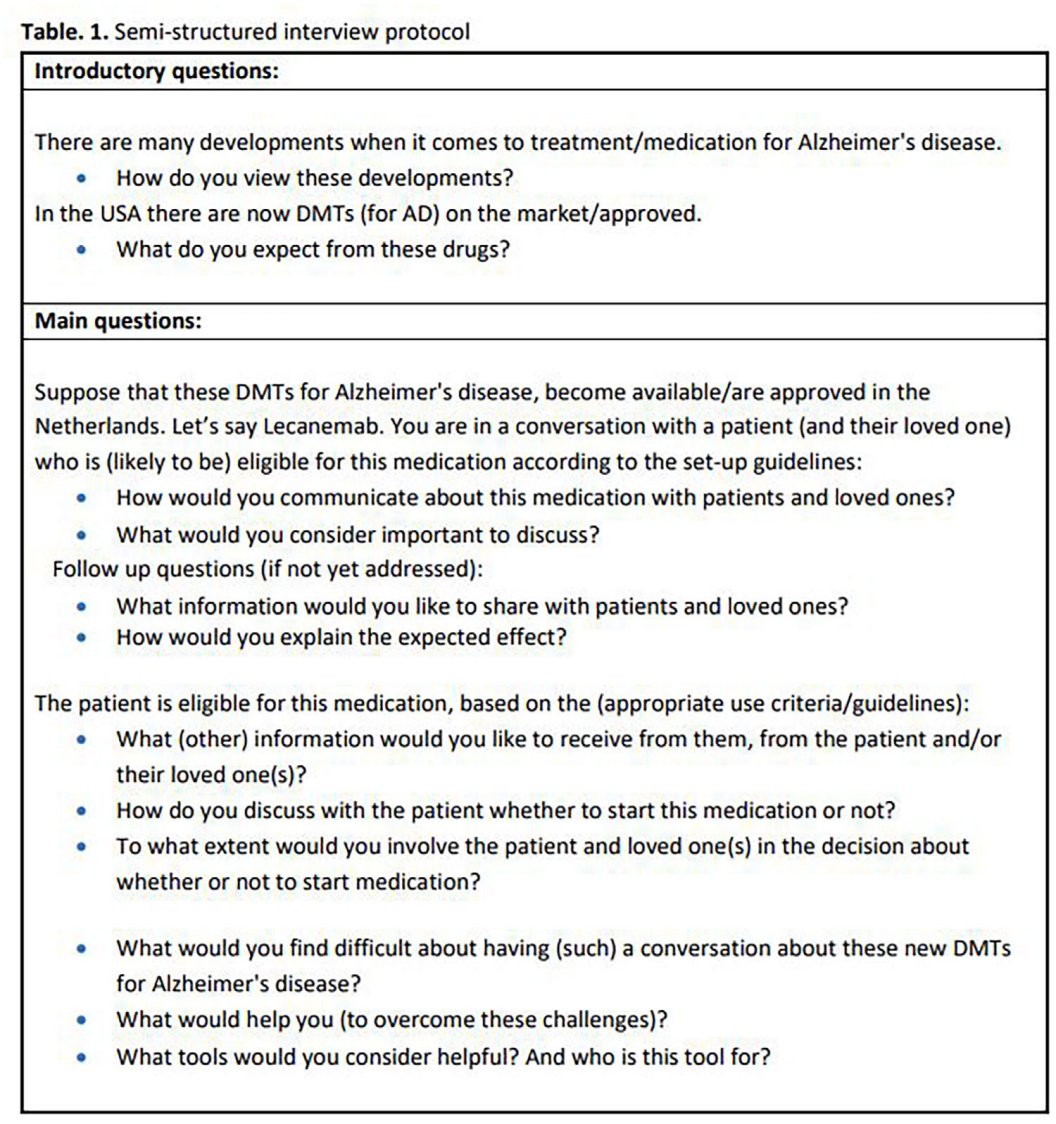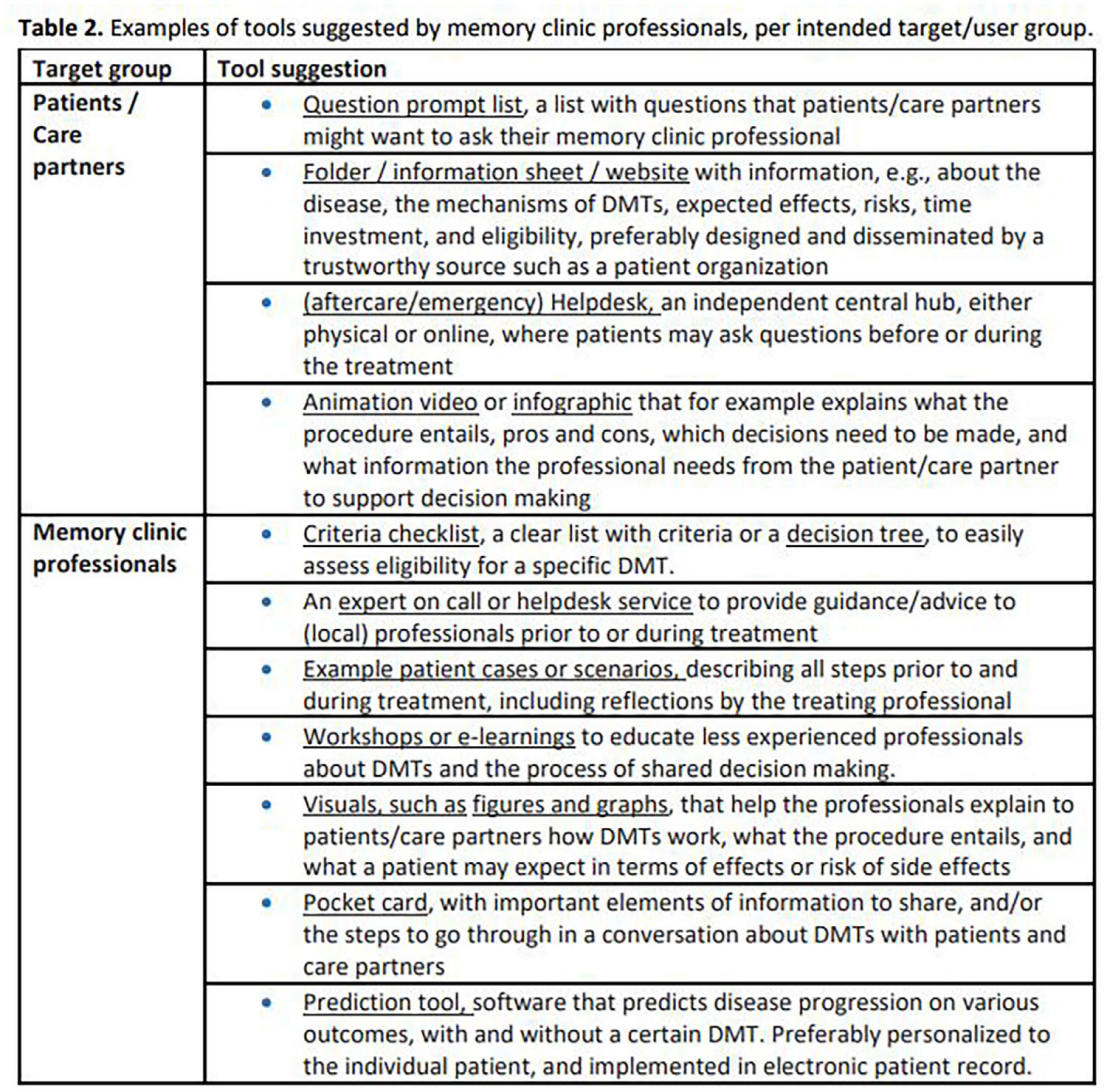# Communicating with patients about the new disease‐modifying treatment(s) for Alzheimer’s disease: the perspective of memory clinic professionals

**DOI:** 10.1002/alz.084012

**Published:** 2025-01-09

**Authors:** Leonie N.C. Visser, Tjeerd Fluitman, Aniek M. van Gils, Pieter J. van der Veere, Argonde C. van Harten, Everard G.B. Vijverberg, Wiesje M. van der Flier

**Affiliations:** ^1^ Medical Psychology, Amsterdam UMC location AMC, University of Amsterdam, Amsterdam Netherlands; ^2^ Amsterdam Public Health, Quality of Care, Personalized Medicine, Amsterdam Netherlands; ^3^ Amsterdam Neuroscience, Neurodegeneration, Amsterdam Netherlands; ^4^ Division of Clinical Geriatrics, Center for Alzheimer Research, Department of Neurobiology, Care Sciences and Society, Karolinska Institutet, Stockholm Sweden; ^5^ Alzheimer Center Amsterdam, Neurology, Vrije Universiteit Amsterdam, Amsterdam UMC location VUmc, Amsterdam Netherlands; ^6^ Amsterdam UMC, Amsterdam, Noord‐holland Netherlands; ^7^ Alzheimer Center Amsterdam, Department of Neurology, Amsterdam Neuroscience, Vrije Universiteit Amsterdam, Amsterdam UMC, Amsterdam Netherlands; ^8^ Department of Epidemiology and Data Science, Amsterdam UMC, Amsterdam Netherlands; ^9^ Amsterdam Neuroscience, Vrije Universiteit Amsterdam, Amsterdam UMC, Amsterdam Netherlands; ^10^ Department of Epidemiology and Data Science, Vrije Universiteit Amsterdam, Amsterdam UMC, Amsterdam Netherlands; ^11^ Alzheimer Center Amsterdam, Neurology, Vrije Universiteit Amsterdam, Amsterdam UMC, Amsterdam Netherlands

## Abstract

**Background:**

The first disease‐modifying treatments (DMTs) for Alzheimer’s disease (AD) have been approved in the USA, marking profound changes in AD‐diagnosis and treatment. This will bring new challenges in terms of clinician‐patient communication. We aimed to collect the perspectives of memory clinic professionals regarding the most important topics to address and what (tools) would support professionals and their patients and care partners to engage in a meaningful conversation on whether (or not) to initiate treatment.

**Methods:**

Semi‐structured interviews were conducted with 16 professionals (10 neurologists, 4 geriatricians, 2 other; 7 female) with >5 years of experience in various Dutch memory clinics. We described the context of (at the time hypothetical) EMA approval, and asked questions such as ‘How would you communicate with patients about DMTs?’(Table 1). The transcribed audio‐recordings were analyzed by means of thematic analysis using MaxQDA software.

**Results:**

A first overarching theme concerned the ambivalence that professionals expressed towards DMTs. On the one hand, they considered approval of the first DMTs as a hopeful development. On the other hand, they expressed caution considering expected effects, risks of (serious) side effects, and burdensome procedures. Professionals therefore emphasized the need for shared decision‐making (SDM) with patients and care partners. Second, to enable SDM, they stressed that professionals and patients must comprehend essential information, including the mechanisms of AD and DMTs, benefits, and potential negative aspects, and that patients/care partners express their needs, expectations, and personal circumstances. Third, professionals anticipated challenges, including the required consultation time and insufficient knowledge/experience with DMTs and/or SDM. They would therefore value education and communication tools, also for patients and care partners, to help them prepare for consultations and increase understanding (Table 2).

**Conclusion:**

A broad representation of memory clinic professionals emphasized that initiating DMT for AD is a preference‐sensitive decision. SDM is not a new concept in memory clinics, yet professionals considered SDM imperative and more complex in the DMT‐context, because of the uncertainties regarding effects, risk of side effects, and burden of DMT‐administration/monitoring. To support professionals and patients in having a meaningful conversation about DMT‐initiation, educational materials and communication tools are needed.